# *Neurospora discreta* as a model to assess adaptation of soil fungi to warming

**DOI:** 10.1186/s12862-015-0482-2

**Published:** 2015-09-16

**Authors:** Adriana L. Romero-Olivares, John W. Taylor, Kathleen K. Treseder

**Affiliations:** Department of Ecology and Evolutionary Biology, University of California-Irvine, Irvine, CA 92697-2525 USA; Department of Plant and Microbial Ecology, 111 Koshland Hall, University of California-Berkeley, Berkeley, CA 94720-3102 USA

## Abstract

**Background:**

Short-term experiments have indicated that warmer temperatures can alter fungal biomass production and CO_2_ respiration, with potential consequences for soil C storage. However, we know little about the capacity of fungi to adapt to warming in ways that may alter C dynamics. Thus, we exposed *Neurospora discreta* to moderately warm (16 °C) and warm (28 °C) selective temperatures for 1500 mitotic generations, and then examined changes in mycelial growth rate, biomass, spore production, and CO_2_ respiration. We tested the hypothesis that strains will adapt to its selective temperature. Specifically, we expected that adapted strains would grow faster, and produce more spores per unit biomass (i.e., relative spore production). In contrast, they should generate less CO_2_ per unit biomass due to higher efficiency in carbon use metabolism (i.e., lower mass specific respiration, MSR).

**Results:**

Indeed, *N. discreta* adapted to warm temperatures, based on patterns of relative spore production. Adapted strains produced more spores per unit biomass than parental strains in the selective temperature. Contrary to our expectations, this increase in relative spore production was accompanied by an increase in MSR and a reduction in mycelial growth rate and biomass, compared to parental strains.

**Conclusions:**

Adaptation of *N. discreta* to warm temperatures may have elicited a tradeoff between biomass production and relative spore production, possibly because relative spore production required higher MSR rates. Therefore, our results do not support the idea that adaptation to warm temperatures will lead to a more efficient carbon use metabolism. Our data might help improve climate change model simulations and provide more concise predictions of decomposition processes and carbon feedbacks to the atmosphere.

**Electronic supplementary material:**

The online version of this article (doi:10.1186/s12862-015-0482-2) contains supplementary material, which is available to authorized users.

## Background

It has been proposed that global warming will enhance metabolic activities of microbes, and in doing so, provide a positive feedback to global warming due to high rates of CO_2_ production [1]. Nevertheless, studies have shown contrasting results regarding the response of soil microbes to warming and C cycle feedbacks (e.g. ref [2–5]). Moreover, little is known about the capacity of fungi to evolve in response to warm conditions, and the potential consequences for the C cycle. Given the relatively short generation time of some fungi (*e.g. Neurospora* spp., ref 6) it is possible that they can adapt to warming at ecologically-relevant timescales; recent studies support this idea (e.g. ref [6]). However, the specific changes in physiology and function of adapted fungi remain unknown.

We can examine evolutionary responses of fungi to warming by conducting selection experiments on a model fungus with a particularly fast generation time. This approach should provide information that we can then compare with community and ecosystem level observations [7]. Toward this end, we selected the renowned model fungus *Neurospora discreta*, because it is a globally-distributed saprotrophic fungus that persists in natural fungal communities [8–12]. Moreover, it is readily manipulated under laboratory conditions, where it can complete thousands of generations within a few months [13].

Laboratory studies on individual fungal species have already improved our knowledge regarding physiological responses to climate. For example, experimental work with *Neurospora crassa* showed that this fungus can acclimate to changes in temperature, suggesting that fungi can respond to seasonal changes and to different climates [14]. Additionally, previous research using *N. crassa* [14] and *Saccharomyces cerevisiae* [15] showed that under short-term temperature stress, fungi increase their metabolic activity (core metabolism) and reduce their growth. It also showed that yeasts and filamentous fungi can acclimate quickly to warming, reaching a steady-state which cannot be reverted [16]. In contrast to these acclimation experiments, adaptation has rarely been specifically addressed. In our work, we examined the adaptation of *N. discreta* to warm temperatures. We chose three strains of *N. discreta* that had been isolated from relatively cool environments, and exposed them to 16 °C and 28 °C for 1500 mitotic generations. We then assessed changes in four physiological traits: mycelial growth rate (MGR), relative spore production (i.e., spores per unit biomass), mass specific respiration (MSR), and biomass production. To date, there is no standard measure of fungal fitness [17]. We selected relative spore production as an indicator of reproductive fitness, because it has been suggested that high fitness in saprotrophic fungi is associated with the ability to quickly colonize new environments by allocating resources to spore production [17, 18]. In addition, we used MGR as another fitness trait [18], because fast mycelial growth could also improve colonization ability.

We also examined mass specific respiration (MSR) as a general measure of the efficiency with which fungi use carbon sources, as previously suggested [19]. MSR has been widely used by soil microbial ecologists as a proxy to measure adaptation (e.g. ref [4, 5, 20]) and quantify carbon use efficiency. It has been hypothesized that carbon use efficiency will be greater as microorganisms adapt to warmer temperatures (i.e. higher microbial biomass accompanied by lower MSR); several studies support this idea to some extent (e.g. [2, 21]), but more recent studies have predicted that carbon use efficiency will decline with increasing temperature (e.g. [22]).

We performed these selection experiments to address the question: “What physiological changes coincide with fungal adaptation to warming?” We hypothesized that the adapted strains will grow faster (Hypothesis 1) and have higher relative spore production (Hypothesis 2), than the parental strains in the selective temperature. In addition, we expected lower MSR by the adapted strains than the parental strains in the selective temperature, owing to more efficient carbon use metabolism (Hypothesis 3).

## Results

Our adaptation experiments consisted of exposing three different strains of *N. discreta* to moderately warm (16 °C) and warm (28 °C) temperatures for 1500 generations. These strains were originally isolated from Tok, Alaska; Perma, Montana; and Wells, Nevada (Table 1) [9]. These sites were relatively cool, with mean annual temperatures ranging from −4.7 °C to 8.1 °C (Table 1). The strains grew along 30 cm race tubes while exposed to the selective temperature. We inoculated one end of the race tube with an initial population size of 5 million spores. When the strains had reached the opposite side of the tube (i.e. that 100 % of the area was colonized), we took the last 15 × 20 mm strip of culture (about 5 million spores, and 25 million nuclei) and transferred it to a new tube. These transfers continued until the strains had crossed 15 race tubes, for a total of 450 cm. Prior to starting the adaptation experiment, we assessed MGR, biomass production, relative spore production, and MSR of the parental strains at incubation temperatures ranging from 4 to 28 °C. After the adaptation regime was completed, we performed the same assessment on the 16 °C-adapted and 28 °C-adapted strains.

**Table 1:** Location and climatic characteristics of the wild isolates of *Neurospora discreta* used in this work

Location	Geographic coordinates^a^	Altitude (masl)^a^	Mean annual precipitation (mm y^−1^)^b^	Mean annual temperature (°C)^b^	FGSC id
Tok, AK	63° 21′ N, 142° 60′ W	515	234	−4.7	9979
Perma, MT	47° 23′ N, 114° 35′ W	930	351	7.5	8572
Wells, NV	41° 12′ N, 114° 57′ W	1952	248	8.1	8565

^a^From Jacobson *et al.* 2004

^b^Data from Western Regional Climate Center, 2014, wrcc.dri.edu

### Parental strains

**Fig. 1:**
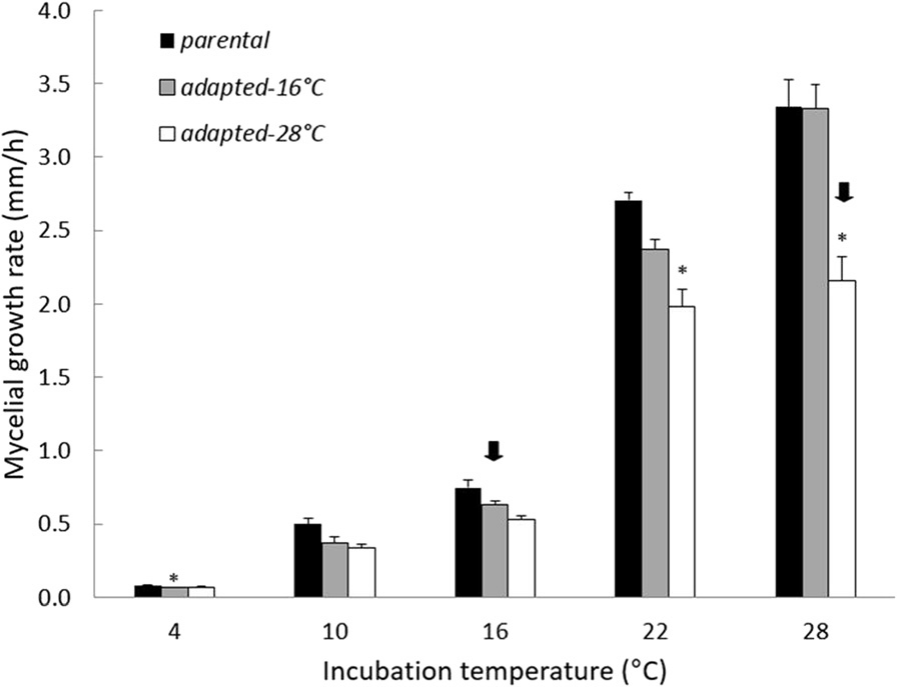
Mycelial growth rate of parental and adapted strains of *N. discreta* measured at different incubation temperatures. Bars are means of all three geographical strains and their replicates + 1SE (*n =* 9). Asterisks indicate significant pairwise differences between adapted and parental strains within a given incubation temperature (*P <* 0.05). Arrows indicate the selective temperature for each adapted strain.

**Fig. 2:**
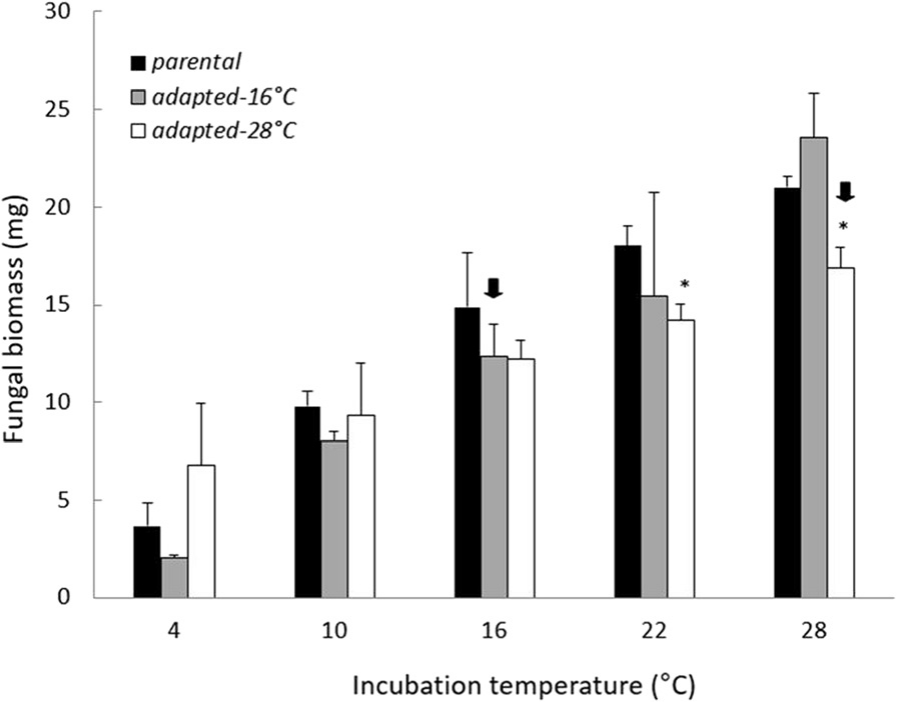
Fungal biomass of parental and adapted strains of *N. discreta* measured at different incubation temperatures. Bars are means of all three geographical strains and their replicates + 1SE (*n =* 9). Asterisks indicate significant pairwise differences between adapted and parental strains within a given incubation temperature (*P <* 0.05). Arrows indicate the selective temperature for each adapted strain.

**Fig. 3:**
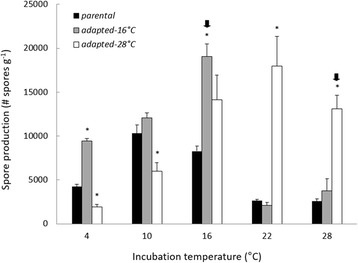
Spore production of parental and adapted strains of *N. discreta* measured at different incubation temperatures. Bars are means of all three geographical strains and their replicates + 1SE (*n =* 9). Asterisks indicate significant pairwise differences between adapted and parental strains within a given incubation temperature (*P <* 0.05). Arrows indicate the selective temperature for each adapted strain.

**Fig. 4:**
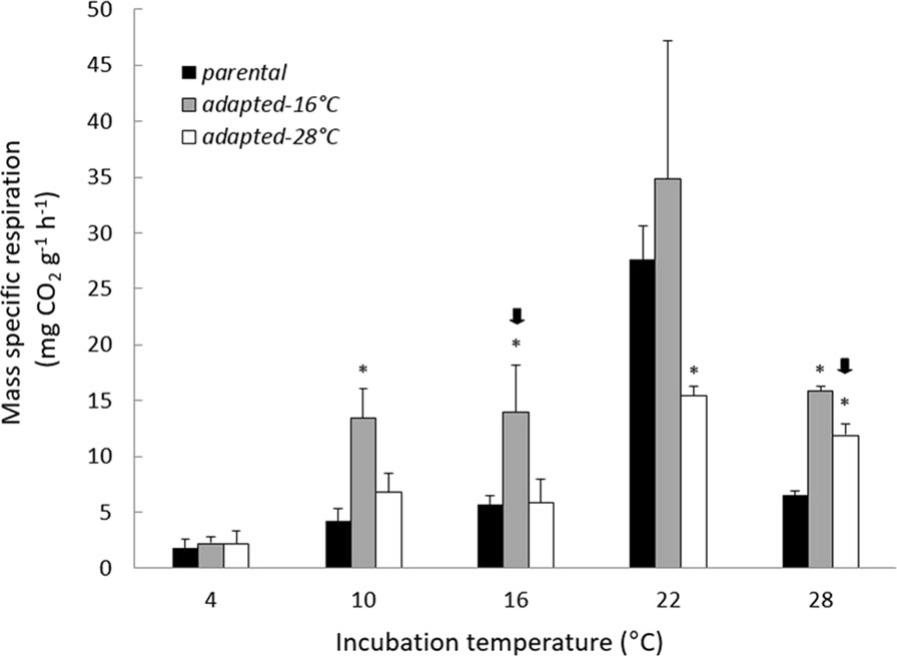
Mass specific respiration of parental and adapted strains of *N. discreta* measured at different incubation temperatures. Bars are means of all three geographical strains and their replicates + 1SE (*n =* 9). Asterisks indicate significant pairwise differences between adapted and parental strains within a given incubation temperature (*P <* 0.05). Arrows indicate the selective temperature for each adapted strain.

We incubated parental strains for 48 h at 4, 10, 16, 22, and 28 °C, and then measured MGR, biomass production, relative spore production, and MSR; they grew faster as incubation temperature increased—a pattern typical of parental and adapted strains alike (Figs. 1 and 2, temperature effect MGR: F_4,8_ = 190.87, *P <* 0.001; temperature effect biomass: F_4,8_ = 420.94, *P <* 0.001). In contrast, relative spore production by the parental strains peaked at 10 °C (Fig. 3), the incubation temperature closest to the mean annual temperatures of the Montana and Nevada sites. Regardless of adaptation status, relative spore production displayed a unimodal-shaped relationship to incubation temperature (Fig. 3, temperature effect, F_4,8_ = 141.87, *P <* 0.001). MSR of the parental strains remained consistently low at the three coolest temperatures, increased three-fold at 22 °C, and declined at the highest temperature (Fig. 4). Indeed, increasing temperatures (up to 22 °C) augmented MSR in parental as well as adapted strains (Fig. 4, temperature effect, F_4,8_ = 20.87, *P <* 0.001).

Overall, the parental strains appeared to be better adapted to cooler temperatures (10 and 16 °C) based on patterns of relative spore production (Fig. 3). MGR, biomass and MSR were sensitive to incubation temperature as well—they all increased markedly at higher temperatures, with the exception of MSR at 28 °C (Figs. 1, 2, and 3). How did the warm-adapted strains compare?

### 16 °C-adapted strains

Contrary to our expectations, MGR of the 16 °C-adapted strains was not higher than parental strains when both were incubated at 16 °C (Fig. 1). Neither was biomass (Fig. 2). In fact, across all incubation temperatures, we observed a significant interaction between adaptation state and incubation temperature (MGR F_9,16_ = 3.4, *P <* 0.001; biomass: F_9,16_ = 310.98, *P <* 0.001), but not in the expected direction at 16 °C. Instead, there was a trend toward a decline in MGR at 16 °C. With respect to the 16 °C adapted strains, we rejected Hypothesis 1, that adapted strains would have higher MGR than parental strains in the selective temperature.

Nevertheless, Hypothesis 2, which predicted that adapted strains would produce more spores per unit biomass compared to parental strains in the selective temperature, was supported. Specifically, 16 °C-adapted strains produced more spores per unit biomass than the parental strains at 16 °C (Fig. 3, *P <* 0.001). Moreover, relative spore production of the 16 °C-adapted strains peaked at 16 °C—at a warmer temperature than did the parental strains (Fig. 3). Accordingly, the interaction between adaptation state and incubation temperature was significant (F_9,16_ = 86.83, *P <* 0.001).

Unexpectedly, MSR of 16 °C-adapted strains was significantly higher than that of parental strains in the selective temperature (Fig. 4, *P =* 0.005). This finding was contrary to Hypothesis 3, which predicted the opposite. The interaction between adaptation state and incubation temperature was significant (Fig. 4; F_9,16_ = 24.33, *P <* 0.001).

In summary, the 16 °C-adapted strains appeared to display higher fitness at 16 °C, in terms of relative spore production (Fig. 3). However, this adaptation was not accompanied by an increase in MGR and biomass (Fig. 1 and 2) as previously hypothesized. Additionally, this adaptation was also accompanied by an increase of MSR rather than a decrease. Did strains adapted to an even warmer temperature display similar physiological shifts?

### 28 °C-adapted strains

The 28 °C-adapted strains displayed physiological shifts that were very similar to the 16 °C-adapted strains (adaptation state*incubation temperature effect MGR: F_9,16_ = 178.87, *P <* 0.001; biomass: F_9,16_ = 379.26, *P <* 0.001). Specifically, MGR and biomass production by the 28 °C-adapted strains was significantly smaller than that of the parental strains when both were incubated at 28 °C (Fig. 1 and 2, MGR *P <* 0.001; biomass: *P <* 0.001). Furthermore, relative spore production at 28 °C increased significantly compared to the parental strain (Fig. 3, *P <* 0.001). We note that relative spore production by the 28 °C-adapted strain tended to peak at the 22 °C incubation temperature, but was not significantly different from the 28 °C incubation temperature (*P =* 0.91). Finally, MSR of the 28 °C-adapted strain was higher than the parental strain at 28 °C (Fig. 4, *P <* 0.001). Accordingly, Hypothesis 2 was supported for adaptation to 28 °C, but not Hypotheses 1 and 3. Similar to the 16 °C-adapted strains, adaptation to 28 °C was accompanied by a reduction in MGR and biomass, as well as an increase in MSR.

### Geographic origin

Strains isolated from the three field sites varied significantly in MGR, biomass, relative spore production, and MSR. Nevertheless, there was no noticeable relationship between mean annual temperature at the site of origin versus biomass, MGR, MSR, or relative spore production.

### Mycelial growth rate versus biomass

Biomass and mycelial growth rate were each of interest in this study. We measured mycelial growth rate as a potential indicator of fitness (in addition to relative spore production). We quantified biomass because it was required to calculate relative spore production and MSR. In addition, microbial biomass is a common component of ecosystem models of soil dynamics [23]; the biomass data from this study can be used by modelers for parameterization or validation. Mycelial growth rate and biomass were strongly -but not perfectly- correlated (Additional file 1: Fig. S1; Spearman’s rank-order correlation ρ = 0.75, *P <* 0.001).

## Discussion

After adaptation to 16 and 28 °C, *N. discreta* displayed an enhanced metabolic rate at those temperatures, perhaps in order to support higher production rates of energetically-expensive spores. Accordingly, tradeoffs in resource allocation may have led to the slower MGR (Fig. 1) and lower biomass production (Fig. 2) in the adapted strains. We rejected Hypothesis 1, because MGR in the warm-adapted strains was not higher at the selective temperature, compared to parental strains (Fig. 1). Nevertheless, Hypothesis 2 was supported because adapted strains of *N. discreta* produced significantly more spores per unit biomass at the selective temperature (Fig. 3). Finally, we rejected Hypothesis 3, because adapted strains produced higher MSR at the selective temperature (Fig. 4), suggesting that adaptation did not lead to greater efficiency of carbon use.

Higher relative spore production in the adapted strains may indicate greater fitness at the selective temperatures. Similarly, in experiments carried out using *Aspergillus niger*, the rate of spore production was used as a measure of fitness. In this case however, colony surface area was used as a unit of biomass, and contrary to our observations, there was a positive trend between biomass and number of spores [24]. More recently, mathematical models have shown that for asexual fungi in nature, higher production of spores facilitates the extension of the colony while avoiding the risk of extending the mycelium into a resource-poor area [18]. In addition, previous studies have reported that fungi that invest in dispersal structures (i.e. spores) instead of vegetative structures (i.e. hyphae) tend to colonize more litter patches and are more prevalent within the ecosystem [25].

The adapted strains of *N. discreta* may have grown more slowly because they were allocating a greater proportion of resources to spore production instead of biomass. Dettman and colleagues [13] also performed temperature-selection experiments with *Neurospora* spp., but they adapted the strains to a cooler temperature of 12 °C. Their cold-adapted strains exhibited an increase in MGR at 12 °C, compared to the parental strains. However, the authors discussed that their method of propagation of lineages (transferring the fastest-growing hyphal tips) might not be the best approach to allow efficient competition and adaptive response [13]. In fact, transferring the fastest growing mycelial sector has been identified as artificial selection, whereas transferring of random samples of mycelial-produced spores has been identified as a more accurate way of replicating natural selection [26]. In our work, we transferred mycelia and spores together, rather than only the fastest growing hyphal tips.

The increase in MSR at high temperatures following adaptation was unexpected. This result might be explained by the increased production of spores in adapted strains (Fig. 2). Spores are known to have energetically-costly compounds such as nutrients for survival during dormancy and complex structural molecules for efficient spore dispersal. For example: the fibrous layer of “rodlets” in the surface of *Neurospora’s* spores make them highly hydrophobic and readily dispersible through air; these compounds are not present in vegetative mycelium and are composed mostly of hydrophobic proteins [27, 28]. In addition, studies in *Penicillium chrysogenum*, showed that production of spores is usually accompanied by thickening of the cell wall of the hyphae and reduced growth rate due to extensive vacuolation and plugging of the hyphal septum; these processes are metabolically costly In fact, a positive relationship between production of spores and MSR has been reported in previous studies [29].

Our findings contrast with those often observed in short-term acclimation experiments. Typically, MSR declines following acclimation of microbes to higher temperatures, although underlying mechanisms are a matter of debate. For example, Luo and collaborators [2] observed a decrease in soil respiration as temperature increased. They suggested that this response was the result of acclimatization to warming by microbes, and concluded that acclimatization might weaken the positive feedbacks to global warming. However, Kirschbaum [3] argued that the apparent acclimatization was due to depletion of labile carbon in the soil. Later, Bradford and collaborators [4] indicated that reduced MSR from microbes resulted from both mechanisms operating in concert. The observations of Schindlbacher and collaborators [5] were an exception; they found that acclimation to warming enhanced MSR. Crowther and Bradford [6] conducted one of the few studies that have assessed the temperature acclimation of fungal species, instead of the microbial community as a whole. They observed an increase in MSR and declines in growth efficiency at elevated temperatures (28 °C) after 10 days of incubation. Since these studies were generally short-term, the fungi likely had minimal opportunity for adaptation.

The response of MSR to temperature in our adaptation experiment may have differed from that of the majority of the acclimation experiments because our experiment selected for higher relative spore production. Sporulation was likely to be less important in shorter-term studies with fewer generation cycles. In ecosystems exposed to global warming, selection for high sporulation rates may lead to increases in MSR at the evolutionary time scale, even though MSR may initially decline owing to short-term acclimation.

Evolution of natural populations occurs by many processes such as gene flow, genetic drift, sexual recombination, and mutation. Those natural processes were not necessarily replicated in our study. The adaptation process that we carried out was performed under controlled conditions, and the sample size for each transfer was kept as consistent as possible. In addition, we focused on one fungal species, so it remains unknown whether similar adaptive responses will occur in other species. Nevertheless, we incorporated some genotypic variation in our study—we used strains of *N. discreta* that were collected from three different sites and that varied initially in spore production and biomass (Table 2). Moreover, for asexually reproducing strains of fungi, selection is easily affected by changes in temperature, because the direction of selection is strongly dependent upon changes in its environment, and not on sexual recombination [30]. Therefore, our results should not be affected by the lack of sexual reproduction.

**Table 2:** Physiological profile of strains from different geographic locations, averaged across incubation temperatures and adaptation state (means ± SE)

Site of origin	Mycelial growth rate (mm/h)	Fungal biomass (mg)	Spore production (# g^−1^)	Mass specific respiration (mg g^−1^ h^−1^)
AK	1.35 ± 0.22	9.25 ± 1.50	6420 ± 521	7 ± 1
MT	1.09 ± 0.18	6.58 ± 1.32	11141 ± 1055	18 ± 3
NV	1.40 ± 0.18	9.96 ± 1.68	7957 ± 538	9 ± 1
	F_2,8_ = 6.28	F_2,8_ = 7.64	F_2,8_ = 7.35	F_2,8_ = 11.36
	*P =* 0.002	*P <* 0.001	*P <* 0.001	*P <* 0.001

Temperature has a direct effect on microbial physiological processes that control decomposition [1]. For example, Allison and collaborators [31] simulated changes in soil carbon under global warming under three scenarios: MSR increases markedly with temperature; MSR increases with temperature, but only moderately; and MSR does not vary with temperature. Their model predicts that soil carbon losses are greatest when MSR remains constant, and smallest when MSR is most sensitive to temperature. This pattern occurs because high MSR leads to low microbial biomass and slower ecosystem-level CO_2_ efflux. Our results are most in line with the first scenario, in which MSR increases in response to warming and is particularly sensitive to temperature following adaptation. This pattern may also be true for wild populations of *Heterobasidium parviporum* (a root-rot pathogenic fungus), since the annual respiration activity of this fungus is increasing with annual air temperature in boreal ecosystems of northern Finland [32]. Based on the modeled predictions of Allison and collaborators [31], we might expect that the adaptation responses we observed in *N. discreta* would lead to a mitigation of soil carbon loss. If this response were widespread globally, it might slow the enrichment of atmospheric CO_2_ under warmer conditions. Nevertheless, it remains to be seen whether other fungi will adapt to warming in similar ways.

## Conclusions

In conclusion, our data show that fungi can adapt to warm temperatures. This adaptation might be accompanied by evolutionary tradeoffs such as increased allocation of resources to spore production but reduced MGR and higher MSR. Our results provide little support for the idea that adaptation to global warming will lead to increases in carbon use efficiency. Incorporating this information to climate change model simulations could help provide a more concise forecast on decomposition processes and carbon feedbacks to the atmosphere.

## Methods

### Strains

We selected three strains of *N. discreta* isolated in 2000 and 2001 from AK (Tok, Alaska, FGSC 9979), MT (Perma, Montana, FGSC 8572), and NV (Wells, Nevada, FGSC 8565) [9]. Mean annual temperatures at these sites is −4.7 °C in Alaska, 7.5 °C in Montana and 8.1 °C in Nevada (Table 1). In addition, the mean annual precipitation at all sites was low (234 to 351 mm y^−1^). Strains were maintained on VMM agar (Vogel’s Minimum Medium: 1x Vogel’s salt solution, 1.5 % sucrose, 1.5 % agar).

### Experimental design

For each parental strain (AK, MT, and NV), we conducted three adaptations to 16 °C and another three to 28 °C. The selection experiment lasted 1500 mitotic generations. For each parental strain, we used these 3 × 16 °C adapted and 3 × 28 °C adapted strains plus 3 replicates of the parental strain to assess physiological traits at five temperatures (4, 10, 16, 22, 28 °C). The four measures by which we assessed physiological traits were: MGR, biomass, relative spore production (number of spores per unit biomass), and MSR. For each physiological trait, the total number of data points was 135: 3 geographic regions (AK, MT, NV) * 9 adapted and parental strains (3 × 16 °C + 3 x 28 °C + 3 x parental) * 5 temperatures (4, 10, 16, 22 or 28 °C).

### Inoculum preparation

For all physiological measurements, our inoculum was 5 million spores in 5 ml VMM broth or agar. We prepared the inoculum by adding spores from mycelium (grown on VMM agar) after 5 days of growth, flooding the culture with 20 ml of 1 M sorbitol, filtering the liquid, and recovering the spores by centrifuging for 5 min at 2,500 rpm. The spores were then washed in fresh sorbitol, recovered by centrifugation (3x), and stored at −20 °C. We used a Neubauer chamber to determine the spore concentrations in dilutions of the stored spores.

### Adaptation and physiological tests

We initiated the adaptation process by adding 5 million spores to 20 ml VMM agar in 30 cm race tubes. Based on a total growth of mycelium over 450 cm, we estimate that the adaptation process involved 1500 mitotic generations (briefly, 100 cell cycles are completed every 30 cm of growth based on a cell cycle time of 1.5 h and a mycelial extension rate of ~ 2 mm/h at 22 °C on VMM agar) [13]. To transfer mycelium between race tubes, we collected the last 2 cm of agar with mycelium and spores. The final collection was done in the same manner, except mycelium was transferred to a 50 ml tube and grown for one more week before harvesting spores.

Each physiological test was initiated by adding 5 million spores to 5 ml of VVM agar at 9:00 pm, incubating in darkness for 12 h at 22 °C, and then shifting the culture to the treatment temperature (4, 10, 16, 22 or 28 °C) for an additional 48 h, also in darkness. By standardizing inoculum, germination time, and germination temperature, we could assume that all cultures were at the same life stage when they were shifted to the treatment temperatures.

Spore production was quantified on VMM agar as described for inoculum preparation and reported as the number of spores per g of fungal biomass.

MGR was measured in the selective environment (race tubes with VMM agar) after growth for 48 h at the treatment temperature. MGR was reported as mm per hour on the average of all replicates divided by 48 h of incubation time.

Biomass was determined in VMM agar after growth for 48 h at the treatment temperature by melting the agar (autoclaving at 121 °C for 10 min) and filtering through Whatman #1 filter paper [6]. We then dried the mycelium for 48 h at 60 °C and weighed it to determine fungal biomass (mg).

MSR was measured in VMM agar (same samples used to determine biomass) using septum vials and an infrared gas analyzer (PP Systems EGM-4, Amesbury, MA, USA). After 48 h of incubation at the treatment temperature, we equilibrated gas concentration of all samples by opening the vials under a laminar flow hood for approximately 15 min. We proceeded to close the vials tightly and then incubated the samples for four more hours. We measured CO_2_ concentrations in ppm before and after the four-hour incubation and, by calculating the difference in CO_2_ concentration between the two time points and dividing by biomass, we could calculate MSR as mg CO_2_ g^−1^ fungal biomass h^−1^.

### Statistical analyses

For each physiological test, we conducted a nested repeated measures analysis of variance (ANOVA). The independent variables were adapted state (parental, 16 °C-adapted, and 28 °C-adapted) and strain origin (AK, MT, and NV), with strain origin nested within adapted state. Incubation temperature was the repeated measure. The dependent variable was spore production, biomass, or MSR. Kolmogorov-Smirnov *post hoc* tests were used to assess pairwise differences. Significant interactions between adapted state and incubation temperature would support our hypotheses if the 16 °C- or 28 °C-adapted strains also displayed significantly higher sporulation, less biomass, and lower MSR than the parental strains when all were incubated at the selective temperature. Differences were considered significant when *P <* 0.05. We ranked all data, because they did not conform to assumptions for normality or homogeneity of variances. All statistical analyses were done using the statistical program R (www.r-project.org) and SYSTAT (SPSS, Evanston, IL).

## Additional file

Additional file 1: Figure S1. Correlation between biomass and mycelial growth rates of parental and adapted strains of *N. discreta* measured at different temperatures (ρ = 0.75, *P <* 0.001). Each symbol represents a different combination of incubation temperature and adapted state (parental, 16 °C-adapted, and 28 °C-adapted). Symbols are means ± 1SE of all three geographical strains and their replicates (*n =* 9).

## Abbreviations

MGR: Mycelial Growth Rate
MSR: Mass Specific Respiration
