# Supplementary figures and images for: Neurospora discreta as a model to assess adaptation of soil fungi to warming
Source: BMC Evol Biol. 2015 Sep 16;15:198. doi: 10.1186/s12862-015-0482-2 (PMC4573461; doi:10.1186/s12862-015-0482-2)

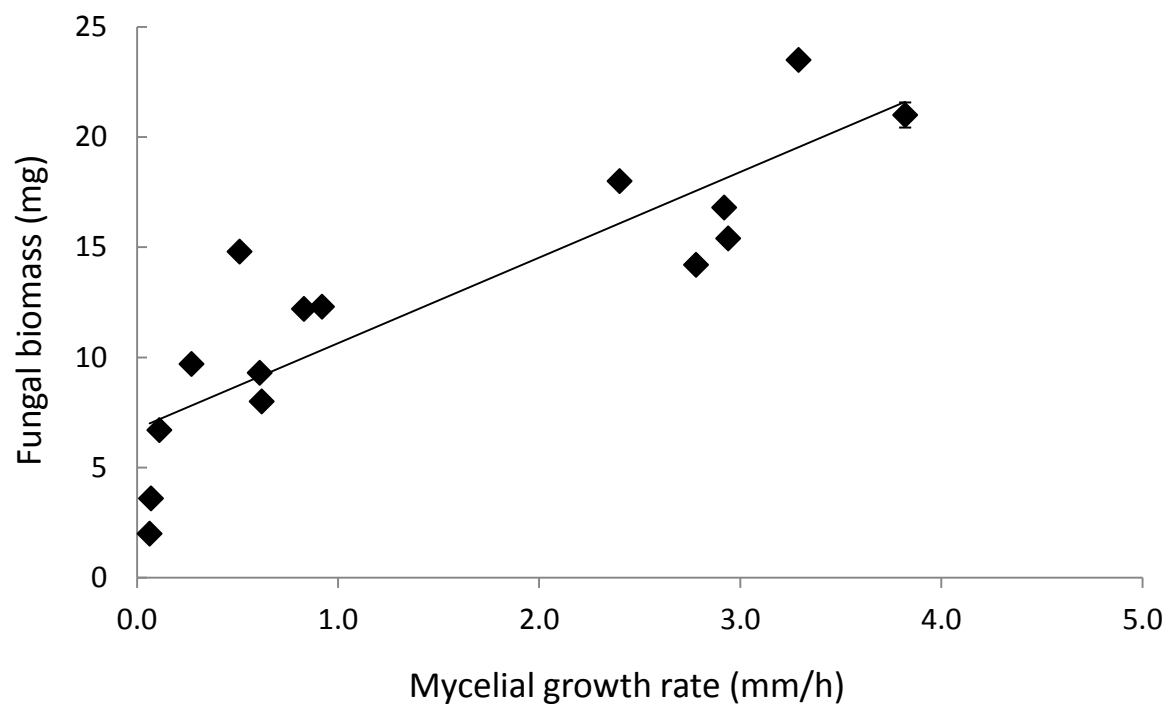

Supplement: Additional file 1: Figure S1. — Correlation between biomass and mycelial growth rates of parental and adapted strains of N. discreta measured at different temperatures (ρ = 0.75, P < 0.001). Each symbol represents a different combination of incubation temperature and adapted state (parental, 16 °C-adapted, and 28 °C-adapted). Symbols are means ± 1SE of all three geographical strains and their replicates (n = 9). [file 12862_2015_482_MOESM1_ESM.pdf]
